# Supplementary material for: Lmx1b is required at multiple stages to build expansive serotonergic axon architectures
Source: eLife. 2019 Jul 29;8:e48788. doi: 10.7554/eLife.48788 (PMC6685705; doi:10.7554/eLife.48788)
Supplement: Supplementary file 1. — Axon genes that are also regulated by Pet1 in rostral 5-HT neurons at E17.5 are in bold. [file elife-48788-supp1.docx]

**Supplementary File 1. Lmx1b-regulated axon-related genes in rostral and caudal 5-HT neurons** **at E17.5.** Axon genes that are also regulated by Pet1 in rostral 5-HT neurons at E17.5 are in bold.

| Gene_name | Gene_description | GO_terms | Rostral (log2fold  change) | Caudal (log2 fold  change) |
| --- | --- | --- | --- | --- |
| 2900011O08Rik | RIKEN cDNA 2900011O08 gene [Source:MGI Symbol;Acc:MGI:1914504] | regulation of supramolecular fiber organization |  | -1.2925 |
| Aak1 | AP2 associated kinase 1 [Source:MGI Symbol;Acc:MGI:1098687] | axon part, cell leading edge |  | -1.34758 |
| Abi1 | abl-interactor 1 [Source:MGI Symbol;Acc:MGI:104913] | axon part, cell leading edge |  | -2.08274 |
| Abi2 | abl-interactor 2 [Source:MGI Symbol;Acc:MGI:106913] | regulation of supramolecular fiber organization, cell leading edge |  | -1.26797 |
| Ablim1 | actin-binding LIM protein 1 [Source:MGI Symbol;Acc:MGI:1194500] | axon development, cell leading edge |  | -0.986226 |
| Ablim3 | actin binding LIM protein family, member 3 [Source:MGI Symbol;Acc:MGI:2442582] | cell leading edge |  | -1.4282 |
| Ache | acetylcholinesterase [Source:MGI Symbol;Acc:MGI:87876] | axon development, extracellular matrix binding |  | -2.97281 |
| Acta2 | actin, alpha 2, smooth muscle, aorta [Source:MGI Symbol;Acc:MGI:87909] | tissue migration, cell leading edge |  | 4.47255 |
| Actb | actin, beta [Source:MGI Symbol;Acc:MGI:87904] | axon development, axon part | -1.03241 | -0.884202 |
| Actg1 | actin, gamma, cytoplasmic 1 [Source:MGI Symbol;Acc:MGI:87906] | axon part |  | -1.43707 |
| Adam17 | a disintegrin and metallopeptidase domain 17 [Source:MGI Symbol;Acc:MGI:1096335] | axon development, tissue migration, cell leading edge |  | 1.88974 |
| Adam9 | a disintegrin and metallopeptidase domain 9 (meltrin gamma) [Source:MGI Symbol;Acc:MGI:105376] | tissue migration, cell-substrate adhesion, extracellular matrix binding |  | 2.6695 |
| Adcyap1 | adenylate cyclase activating polypeptide 1 [Source:MGI Symbol;Acc:MGI:105094] | axon part |  | -1.93698 |
| Add2 | adducin 2 (beta) [Source:MGI Symbol;Acc:MGI:87919] | regulation of supramolecular fiber organization |  | -1.4117 |
| Add3 | adducin 3 (gamma) [Source:MGI Symbol;Acc:MGI:1351615] | regulation of supramolecular fiber organization | -1.16861 |  |
| Aif1l | allograft inflammatory factor 1-like [Source:MGI Symbol;Acc:MGI:1919598] | cell leading edge |  | 3.95673 |
| Ajap1 | adherens junction associated protein 1 [Source:MGI Symbol;Acc:MGI:2685419] | cell-substrate adhesion |  | -1.9163 |
| Alcam | activated leukocyte cell adhesion molecule [Source:MGI Symbol;Acc:MGI:1313266] | axon development | -0.765743 |  |
| Amigo1 | adhesion molecule with Ig like domain 1 [Source:MGI Symbol;Acc:MGI:2653612] | axon development, neuron projection fasciculation |  | -2.04702 |
| Amot | angiomotin [Source:MGI Symbol;Acc:MGI:108440] | tissue migration, cell leading edge |  | 2.51561 |
| Ap1ar | adaptor-related protein complex 1 associated regulatory protein [Source:MGI Symbol;Acc:MGI:2384822] | regulation of supramolecular fiber organization, cell-substrate adhesion | -0.612637 | -1.42124 |
| Ap1s1 | adaptor protein complex AP-1, sigma 1 [Source:MGI Symbol;Acc:MGI:1098244] | axon part |  | -2.79248 |
| Ap3b1 | adaptor-related protein complex 3, beta 1 subunit [Source:MGI Symbol;Acc:MGI:1333879] | axon part | -1.06225 |  |
| Ap3m2 | adaptor-related protein complex 3, mu 2 subunit [Source:MGI Symbol;Acc:MGI:1929214] | axon part | -1.12645 |  |
| Ap3s2 | adaptor-related protein complex 3, sigma 2 subunit [Source:MGI Symbol;Acc:MGI:1337060] | axon part |  | -1.67152 |
| Apoe | apolipoprotein E [Source:MGI Symbol;Acc:MGI:88057] | axon development, regulation of supramolecular fiber organization, tissue migration |  | 5.45882 |
| App | amyloid beta (A4) precursor protein [Source:MGI Symbol;Acc:MGI:88059] | axon development, regulation of supramolecular fiber organization, axon part, cell leading edge | -0.667049 |  |
| Arf6 | ADP-ribosylation factor 6 [Source:MGI Symbol;Acc:MGI:99435] | regulation of supramolecular fiber organization, tissue migration, cell leading edge | -0.919736 |  |
| Arhgap31 | Rho GTPase activating protein 31 [Source:MGI Symbol;Acc:MGI:1333857] | cell leading edge |  | 2.69056 |
| Arpc1a | actin related protein 2/3 complex, subunit 1A [Source:MGI Symbol;Acc:MGI:1928896] | regulation of supramolecular fiber organization |  | -1.15811 |
| Arpc1b | actin related protein 2/3 complex, subunit 1B [Source:MGI Symbol;Acc:MGI:1343142] | regulation of supramolecular fiber organization |  | 6.51629 |
| Arpc5 | actin related protein 2/3 complex, subunit 5 [Source:MGI Symbol;Acc:MGI:1915021] | regulation of supramolecular fiber organization, axon part, cell leading edge |  | -1.67729 |
| Arpc5l | actin related protein 2/3 complex, subunit 5-like [Source:MGI Symbol;Acc:MGI:1921442] | regulation of supramolecular fiber organization |  | -1.59761 |
| Arsb | arylsulfatase B [Source:MGI Symbol;Acc:MGI:88075] | tissue migration |  | 1.82901 |
| Asap3 | ArfGAP with SH3 domain, ankyrin repeat and PH domain 3 [Source:MGI Symbol;Acc:MGI:2684986] | regulation of supramolecular fiber organization, cell leading edge |  | 2.39956 |
| Atl1 | atlastin GTPase 1 [Source:MGI Symbol;Acc:MGI:1921241] | axon development | -0.731784 | -1.34143 |
| Atp1a3 | ATPase, Na+/K+ transporting, alpha 3 polypeptide [Source:MGI Symbol;Acc:MGI:88107] | axon part |  | -2.17889 |
| Atp5a1 | ATP synthase, H+ transporting, mitochondrial F1 complex, alpha subunit 1 [Source:MGI Symbol;Acc:MGI:88115] | tissue migration | -0.694647 |  |
| Atp6v0d1 | ATPase, H+ transporting, lysosomal V0 subunit D1 [Source:MGI Symbol;Acc:MGI:1201778] | axon part |  | -1.62577 |
| Atp6v1b2 | ATPase, H+ transporting, lysosomal V1 subunit B2 [Source:MGI Symbol;Acc:MGI:109618] | cell leading edge |  | -1.15726 |
| Atp8a2 | ATPase, aminophospholipid transporter-like, class I, type 8A, member 2 [Source:MGI Symbol;Acc:MGI:1354710] | axon development | -0.733312 |  |
| Axl | AXL receptor tyrosine kinase [Source:MGI Symbol;Acc:MGI:1347244] | cell-substrate adhesion |  | 3.94595 |
| Bcl11b | B cell leukemia/lymphoma 11B [Source:MGI Symbol;Acc:MGI:1929913] | axon development |  | -1.81451 |
| Bcl2l11 | BCL2-like 11 (apoptosis facilitator) [Source:MGI Symbol;Acc:MGI:1197519] | cell-substrate adhesion | -1.505 |  |
| Bdnf | brain derived neurotrophic factor [Source:MGI Symbol;Acc:MGI:88145] | axon development, axon part |  | -3.41876 |
| Bmper | BMP-binding endothelial regulator [Source:MGI Symbol;Acc:MGI:1920480] | tissue migration | 1.7843 |  |
| Bmpr1b | bone morphogenetic protein receptor, type 1B [Source:MGI Symbol;Acc:MGI:107191] | axon development |  | 4.08211 |
| Boc | biregional cell adhesion molecule-related/down-regulated by oncogenes (Cdon) binding protein [Source:MGI Symbol;Acc:MGI:2151153] | axon development, axon part |  | 2.43231 |
| Brsk2 | BR serine/threonine kinase 2 [Source:MGI Symbol;Acc:MGI:1923020] | axon development, axon part | -0.653647 | -1.30374 |
| Btrc | beta-transducin repeat containing protein [Source:MGI Symbol;Acc:MGI:1338871] | morphogenesis of a branching structure | -0.76619 | -1.12134 |
| Cacna1a | calcium channel, voltage-dependent, P/Q type, alpha 1A subunit [Source:MGI Symbol;Acc:MGI:109482] | axon development | -0.67991 | -2.73529 |
| Cacna1b | calcium channel, voltage-dependent, N type, alpha 1B subunit [Source:MGI Symbol;Acc:MGI:88296] | axon part |  | -1.48743 |
| Calm1 | calmodulin 1 [Source:MGI Symbol;Acc:MGI:88251] | axon part |  | -1.16808 |
| Camsap3 | calmodulin regulated spectrin-associated protein family, member 3 [Source:MGI Symbol;Acc:MGI:1916947] | regulation of supramolecular fiber organization, cell-substrate adhesion |  | -1.51755 |
| Capza2 | capping protein (actin filament) muscle Z-line, alpha 2 [Source:MGI Symbol;Acc:MGI:106222] | regulation of supramolecular fiber organization |  | 1.11768 |
| Cask | calcium/calmodulin-dependent serine protein kinase (MAGUK family) [Source:MGI Symbol;Acc:MGI:1309489] | cell-substrate adhesion | -0.587634 |  |
| Cav1 | caveolin 1, caveolae protein [Source:MGI Symbol;Acc:MGI:102709] | regulation of supramolecular fiber organization |  | 4.46864 |
| Cckar | cholecystokinin A receptor [Source:MGI Symbol;Acc:MGI:99478] | axon development, axon part | -2.32076 |  |
| Cd44 | CD44 antigen [Source:MGI Symbol;Acc:MGI:88338] | morphogenesis of a branching structure, cell leading edge |  | 5.56888 |
| Cd47 | CD47 antigen (Rh-related antigen, integrin-associated signal transducer) [Source:MGI Symbol;Acc:MGI:96617] | regulation of supramolecular fiber organization | -0.847659 | -1.62198 |
| Cd63 | CD63 antigen [Source:MGI Symbol;Acc:MGI:99529] | tissue migration, cell-substrate adhesion |  | 1.34976 |
| Cdh13 | cadherin 13 [Source:MGI Symbol;Acc:MGI:99551] | tissue migration, cell-substrate adhesion | -0.980126 |  |
| Cdh2 | cadherin 2 [Source:MGI Symbol;Acc:MGI:88355] | axon development, cell leading edge |  | -1.77896 |
| Cdh4 | cadherin 4 [Source:MGI Symbol;Acc:MGI:99218] | axon development |  | -2.59285 |
| Cdk5 | cyclin-dependent kinase 5 [Source:MGI Symbol;Acc:MGI:101765] | axon development, cell-substrate adhesion, axon part, cell leading edge |  | -1.42201 |
| Cdk5r1 | cyclin-dependent kinase 5, regulatory subunit 1 (p35) [Source:MGI Symbol;Acc:MGI:101764] | axon development, neuron projection fasciculation, axon part |  | -1.31658 |
| Cdk5r2 | cyclin-dependent kinase 5, regulatory subunit 2 (p39) [Source:MGI Symbol;Acc:MGI:1330828] | axon part |  | -3.12076 |
| Cdk5rap2 | CDK5 regulatory subunit associated protein 2 [Source:MGI Symbol;Acc:MGI:2384875] | regulation of supramolecular fiber organization |  | 1.59211 |
| Cdkl3 | cyclin-dependent kinase-like 3 [Source:MGI Symbol;Acc:MGI:2388268] | axon development | -1.06921 |  |
| Celsr1 | cadherin, EGF LAG seven-pass G-type receptor 1 [Source:MGI Symbol;Acc:MGI:1100883] | morphogenesis of a branching structure |  | 2.56377 |
| Celsr3 | cadherin, EGF LAG seven-pass G-type receptor 3 [Source:MGI Symbol;Acc:MGI:1858236] | axon development, neuron projection fasciculation |  | -1.63288 |
| Chn1 | chimerin 1 [Source:MGI Symbol;Acc:MGI:1915674] | axon development | -0.83102 |  |
| **Cited1** | **Cbp/p300-interacting transactivator with Glu/Asp-rich carboxy-terminal domain 1 [Source:MGI Symbol;Acc:MGI:108023]** | **morphogenesis of a branching structure** | **-1.4645** |  |
| Ckap2 | cytoskeleton associated protein 2 [Source:MGI Symbol;Acc:MGI:1931797] | regulation of supramolecular fiber organization |  | 3.29679 |
| Clasp1 | CLIP associating protein 1 [Source:MGI Symbol;Acc:MGI:1923957] | regulation of supramolecular fiber organization, tissue migration, cell-substrate adhesion |  | -1.04159 |
| Clasp2 | CLIP associating protein 2 [Source:MGI Symbol;Acc:MGI:1923749] | axon development, regulation of supramolecular fiber organization, tissue migration, cell-substrate adhesion, axon part, cell leading edge |  | -1.10211 |
| Clcn3 | chloride channel, voltage-sensitive 3 [Source:MGI Symbol;Acc:MGI:103555] | axon part | -0.827676 | -1.05774 |
| Clic4 | chloride intracellular channel 4 (mitochondrial) [Source:MGI Symbol;Acc:MGI:1352754] | morphogenesis of a branching structure |  | 1.6356 |
| Clip1 | CAP-GLY domain containing linker protein 1 [Source:MGI Symbol;Acc:MGI:1928401] | regulation of supramolecular fiber organization, cell leading edge | -0.757095 |  |
| Cnr1 | cannabinoid receptor 1 (brain) [Source:MGI Symbol;Acc:MGI:104615] | axon development, neuron projection fasciculation, axon part |  | -1.31172 |
| Cntn2 | contactin 2 [Source:MGI Symbol;Acc:MGI:104518] | axon development, neuron projection fasciculation, cell-substrate adhesion, axon part |  | -1.37312 |
| Cobl | cordon-bleu WH2 repeat [Source:MGI Symbol;Acc:MGI:105056] | axon development, axon part, cell leading edge |  | -1.66649 |
| Col25a1 | collagen, type XXV, alpha 1 [Source:MGI Symbol;Acc:MGI:1924268] | axon development | -1.2187 | -2.93916 |
| Col4a1 | collagen, type IV, alpha 1 [Source:MGI Symbol;Acc:MGI:88454] | morphogenesis of a branching structure |  | 3.15123 |
| Coro1c | coronin, actin binding protein 1C [Source:MGI Symbol;Acc:MGI:1345964] | tissue migration, cell-substrate adhesion, cell leading edge | -0.656429 | -0.924617 |
| Cpeb1 | cytoplasmic polyadenylation element binding protein 1 [Source:MGI Symbol;Acc:MGI:108442] | axon part |  | -3.17369 |
| Cpeb4 | cytoplasmic polyadenylation element binding protein 4 [Source:MGI Symbol;Acc:MGI:1914829] | axon part |  | -1.56273 |
| Cplx2 | complexin 2 [Source:MGI Symbol;Acc:MGI:104726] | axon part | -0.806513 | -1.11007 |
| Crkl | v-crk avian sarcoma virus CT10 oncogene homolog-like [Source:MGI Symbol;Acc:MGI:104686] | cell-substrate adhesion |  | -1.59903 |
| Crmp1 | collapsin response mediator protein 1 [Source:MGI Symbol;Acc:MGI:107793] | axon development, axon part |  | -1.43007 |
| Crtac1 | cartilage acidic protein 1 [Source:MGI Symbol;Acc:MGI:1920082] | axon development, neuron projection fasciculation, axon part |  | -2.26935 |
| Csnk1e | casein kinase 1, epsilon [Source:MGI Symbol;Acc:MGI:1351660] | axon part |  | -1.50132 |
| Cspg4 | chondroitin sulfate proteoglycan 4 [Source:MGI Symbol;Acc:MGI:2153093] | cell leading edge |  | 2.59491 |
| Ctnnd2 | catenin (cadherin associated protein), delta 2 [Source:MGI Symbol;Acc:MGI:1195966] | morphogenesis of a branching structure |  | -1.05421 |
| Ctsd | cathepsin D [Source:MGI Symbol;Acc:MGI:88562] | morphogenesis of a branching structure |  | 2.17818 |
| Cx3cl1 | chemokine (C-X3-C motif) ligand 1 [Source:MGI Symbol;Acc:MGI:1097153] | regulation of supramolecular fiber organization |  | -2.72845 |
| Cxadr | coxsackie virus and adenovirus receptor [Source:MGI Symbol;Acc:MGI:1201679] | axon part |  | -1.22955 |
| Cxcr4 | chemokine (C-X-C motif) receptor 4 [Source:MGI Symbol;Acc:MGI:109563] | axon development, morphogenesis of a branching structure, axon part, cell leading edge |  | -2.76769 |
| Cyfip1 | cytoplasmic FMR1 interacting protein 1 [Source:MGI Symbol;Acc:MGI:1338801] | axon development, regulation of supramolecular fiber organization, axon part, cell leading edge |  | 1.64498 |
| Cyfip2 | cytoplasmic FMR1 interacting protein 2 [Source:MGI Symbol;Acc:MGI:1924134] | axon development |  | -1.41702 |
| Dab2 | disabled 2, mitogen-responsive phosphoprotein [Source:MGI Symbol;Acc:MGI:109175] | cell-substrate adhesion |  | 6.33366 |
| Dab2ip | disabled 2 interacting protein [Source:MGI Symbol;Acc:MGI:1916851] | tissue migration |  | -1.1787 |
| Dbn1 | drebrin 1 [Source:MGI Symbol;Acc:MGI:1931838] | axon development, regulation of supramolecular fiber organization, cell-substrate adhesion, axon part, cell leading edge |  | -1.5702 |
| Dbnl | drebrin-like [Source:MGI Symbol;Acc:MGI:700006] | regulation of supramolecular fiber organization, cell leading edge |  | -1.479 |
| Dchs1 | dachsous cadherin related 1 [Source:MGI Symbol;Acc:MGI:2685011] | morphogenesis of a branching structure |  | -1.87541 |
| Dclk1 | doublecortin-like kinase 1 [Source:MGI Symbol;Acc:MGI:1330861] | axon development |  | -1.16246 |
| Dlg2 | discs large MAGUK scaffold protein 2 [Source:MGI Symbol;Acc:MGI:1344351] | axon part |  | -1.05242 |
| Dlg4 | discs large MAGUK scaffold protein 4 [Source:MGI Symbol;Acc:MGI:1277959] | axon part |  | -1.66863 |
| Dmd | dystrophin, muscular dystrophy [Source:MGI Symbol;Acc:MGI:94909] | cell-substrate adhesion, cell leading edge |  | 1.1451 |
| Dnm1 | dynamin 1 [Source:MGI Symbol;Acc:MGI:107384] | axon part |  | -1.38927 |
| Dpysl2 | dihydropyrimidinase-like 2 [Source:MGI Symbol;Acc:MGI:1349763] | axon development, axon part | -0.807003 | -1.14169 |
| Dpysl5 | dihydropyrimidinase-like 5 [Source:MGI Symbol;Acc:MGI:1929772] | axon development |  | -2.02166 |
| Draxin | dorsal inhibitory axon guidance protein [Source:MGI Symbol;Acc:MGI:1917683] | axon development |  | 1.33403 |
| Dusp3 | dual specificity phosphatase 3 (vaccinia virus phosphatase VH1-related) [Source:MGI Symbol;Acc:MGI:1919599] | cell-substrate adhesion | -1.01818 |  |
| Edil3 | EGF-like repeats and discoidin I-like domains 3 [Source:MGI Symbol;Acc:MGI:1329025] | cell-substrate adhesion |  | -2.16612 |
| Eef1a1 | eukaryotic translation elongation factor 1 alpha 1 [Source:MGI Symbol;Acc:MGI:1096881] | cell leading edge |  | 1.04097 |
| Efna5 | ephrin A5 [Source:MGI Symbol;Acc:MGI:107444] | axon development, cell-substrate adhesion, negative chemotaxis | -0.842871 | -2.05945 |
| Elavl4 | ELAV like RNA binding protein 4 [Source:MGI Symbol;Acc:MGI:107427] | axon part | -0.702067 | -1.66109 |
| Emc10 | ER membrane protein complex subunit 10 [Source:MGI Symbol;Acc:MGI:1916933] | tissue migration |  | -1.66665 |
| Emp2 | epithelial membrane protein 2 [Source:MGI Symbol;Acc:MGI:1098726] | tissue migration, cell-substrate adhesion |  | 3.7826 |
| Eno2 | enolase 2, gamma neuronal [Source:MGI Symbol;Acc:MGI:95394] | axon part |  | -3.09225 |
| Enpp2 | ectonucleotide pyrophosphatase/phosphodiesterase 2 [Source:MGI Symbol;Acc:MGI:1321390] | tissue migration, cell-substrate adhesion |  | 1.5434 |
| Epha4 | Eph receptor A4 [Source:MGI Symbol;Acc:MGI:98277] | axon development, neuron projection fasciculation, axon part | 1.18643 |  |
| Ephb2 | Eph receptor B2 [Source:MGI Symbol;Acc:MGI:99611] | axon development, neuron projection fasciculation |  | -1.33054 |
| Erc2 | ELKS/RAB6-interacting/CAST family member 2 [Source:MGI Symbol;Acc:MGI:1098749] | axon part | -0.890619 |  |
| Ezr | ezrin [Source:MGI Symbol;Acc:MGI:98931] | cell leading edge |  | 4.12702 |
| Fbxw7 | F-box and WD-40 domain protein 7 [Source:MGI Symbol;Acc:MGI:1354695] | tissue migration | -0.902157 |  |
| Fem1b | feminization 1 homolog b (C. elegans) [Source:MGI Symbol;Acc:MGI:1335087] | morphogenesis of a branching structure | -0.714161 |  |
| **Fgd5** | **FYVE, RhoGEF and PH domain containing 5 [Source:MGI Symbol;Acc:MGI:2443369]** | **cell leading edge** | **-0.980325** | **-2.65118** |
| Fgf13 | fibroblast growth factor 13 [Source:MGI Symbol;Acc:MGI:109178] | regulation of supramolecular fiber organization, axon part | -0.735893 | -1.73578 |
| Fgf18 | fibroblast growth factor 18 [Source:MGI Symbol;Acc:MGI:1277980] | tissue migration | -2.24721 |  |
| **Fkbp1a** | **FK506 binding protein 1a [Source:MGI Symbol;Acc:MGI:95541]** | **axon part** | **-0.834117** | **-2.02553** |
| Fkbp4 | FK506 binding protein 4 [Source:MGI Symbol;Acc:MGI:95543] | regulation of supramolecular fiber organization, axon part |  | -1.53352 |
| Flna | filamin, alpha [Source:MGI Symbol;Acc:MGI:95556] | regulation of supramolecular fiber organization, cell-substrate adhesion, axon part |  | 1.37815 |
| Flrt3 | fibronectin leucine rich transmembrane protein 3 [Source:MGI Symbol;Acc:MGI:1918686] | axon development, negative chemotaxis, axon part |  | -1.93475 |
| Fn1 | fibronectin 1 [Source:MGI Symbol;Acc:MGI:95566] | axon development, cell-substrate adhesion | 2.12294 | 3.25558 |
| Foxp1 | forkhead box P1 [Source:MGI Symbol;Acc:MGI:1914004] | axon development, tissue migration | -1.09187 | -1.3937 |
| Fscn1 | fascin actin-bundling protein 1 [Source:MGI Symbol;Acc:MGI:1352745] | axon part, cell leading edge |  | -2.18864 |
| Gabbr1 | gamma-aminobutyric acid (GABA) B receptor, 1 [Source:MGI Symbol;Acc:MGI:1860139] | axon part, cell leading edge |  | -1.08896 |
| Gabra3 | gamma-aminobutyric acid (GABA) A receptor, subunit alpha 3 [Source:MGI Symbol;Acc:MGI:95615] | cell leading edge |  | -1.69127 |
| Gabra5 | gamma-aminobutyric acid (GABA) A receptor, subunit alpha 5 [Source:MGI Symbol;Acc:MGI:95617] | cell leading edge |  | -1.66817 |
| Gabrb3 | gamma-aminobutyric acid (GABA) A receptor, subunit beta 3 [Source:MGI Symbol;Acc:MGI:95621] | axon part |  | -1.52886 |
| Gabre | gamma-aminobutyric acid (GABA) A receptor, subunit epsilon [Source:MGI Symbol;Acc:MGI:1330235] | cell leading edge |  | -5.67539 |
| Gabrg2 | gamma-aminobutyric acid (GABA) A receptor, subunit gamma 2 [Source:MGI Symbol;Acc:MGI:95623] | cell leading edge |  | -1.49442 |
| Gad1 | glutamate decarboxylase 1 [Source:MGI Symbol;Acc:MGI:95632] | axon part |  | -1.54061 |
| **Gap43** | **growth associated protein 43 [Source:MGI Symbol;Acc:MGI:95639]** | **axon development, axon part** | **-0.708076** | **-2.06642** |
| Gas6 | growth arrest specific 6 [Source:MGI Symbol;Acc:MGI:95660] | cell-substrate adhesion |  | 1.31985 |
| Gata2 | GATA binding protein 2 [Source:MGI Symbol;Acc:MGI:95662] | tissue migration |  | -2.74766 |
| Gata3 | GATA binding protein 3 [Source:MGI Symbol;Acc:MGI:95663] | axon development, tissue migration | -0.664617 | -3.45285 |
| Gbx2 | gastrulation brain homeobox 2 [Source:MGI Symbol;Acc:MGI:95668] | axon development, morphogenesis of a branching structure |  | 3.97612 |
| Git1 | GIT ArfGAP 1 [Source:MGI Symbol;Acc:MGI:1927140] | axon part |  | -2.18834 |
| **Glul** | **glutamate-ammonia ligase (glutamine synthetase) [Source:MGI Symbol;Acc:MGI:95739]** | **tissue migration, axon part** | **-0.72056** | **1.7699** |
| Golga4 | golgi autoantigen, golgin subfamily a, 4 [Source:MGI Symbol;Acc:MGI:1859646] | axon development | 1.3961 |  |
| Gpc1 | glypican 1 [Source:MGI Symbol;Acc:MGI:1194891] | extracellular matrix binding |  | -1.46627 |
| Gpc3 | glypican 3 [Source:MGI Symbol;Acc:MGI:104903] | morphogenesis of a branching structure | -1.68811 |  |
| Gpi1 | glucose phosphate isomerase 1 [Source:MGI Symbol;Acc:MGI:95797] | tissue migration | -0.745484 |  |
| Gpm6a | glycoprotein m6a [Source:MGI Symbol;Acc:MGI:107671] | axon part |  | -1.33119 |
| Gpm6b | glycoprotein m6b [Source:MGI Symbol;Acc:MGI:107672] | cell-substrate adhesion |  | 1.39957 |
| Gria4 | glutamate receptor, ionotropic, AMPA4 (alpha 4) [Source:MGI Symbol;Acc:MGI:95811] | axon part | -1.26559 | -1.35345 |
| Grik5 | glutamate receptor, ionotropic, kainate 5 (gamma 2) [Source:MGI Symbol;Acc:MGI:95818] | axon part | 0.6188 |  |
| Grin1 | glutamate receptor, ionotropic, NMDA1 (zeta 1) [Source:MGI Symbol;Acc:MGI:95819] | axon development, axon part, cell leading edge |  | -2.14365 |
| Grm7 | glutamate receptor, metabotropic 7 [Source:MGI Symbol;Acc:MGI:1351344] | axon part |  | -1.4093 |
| Gsk3b | glycogen synthase kinase 3 beta [Source:MGI Symbol;Acc:MGI:1861437] | axon development, cell-substrate adhesion, axon part | -0.829383 | -1.27975 |
| Hif1a | hypoxia inducible factor 1, alpha subunit [Source:MGI Symbol;Acc:MGI:106918] | tissue migration, axon part | -0.62537 |  |
| Hmox1 | heme oxygenase 1 [Source:MGI Symbol;Acc:MGI:96163] | tissue migration |  | 3.30135 |
| Hsd17b12 | hydroxysteroid (17-beta) dehydrogenase 12 [Source:MGI Symbol;Acc:MGI:1926967] | cell-substrate adhesion |  | 1.3184 |
| Hspa1a | heat shock protein 1A [Source:MGI Symbol;Acc:MGI:96244] | regulation of supramolecular fiber organization |  | 3.94728 |
| Htr1a | 5-hydroxytryptamine (serotonin) receptor 1A [Source:MGI Symbol;Acc:MGI:96273] | regulation of supramolecular fiber organization, axon part | -2.14415 | -3.44427 |
| Igf1 | insulin-like growth factor 1 [Source:MGI Symbol;Acc:MGI:96432] | tissue migration, morphogenesis of a branching structure |  | 5.59491 |
| Igf2 | insulin-like growth factor 2 [Source:MGI Symbol;Acc:MGI:96434] | tissue migration |  | 3.82525 |
| Inpp5f | inositol polyphosphate-5-phosphatase F [Source:MGI Symbol;Acc:MGI:2141867] | axon development |  | -0.926979 |
| Islr2 | immunoglobulin superfamily containing leucine-rich repeat 2 [Source:MGI Symbol;Acc:MGI:2444277] | axon development | 1.4986 | -2.69087 |
| Itga6 | integrin alpha 6 [Source:MGI Symbol;Acc:MGI:96605] | cell-substrate adhesion, extracellular matrix binding |  | 3.42505 |
| Itgav | integrin alpha V [Source:MGI Symbol;Acc:MGI:96608] | tissue migration, cell-substrate adhesion, negative chemotaxis, cell leading edge, extracellular matrix binding |  | 2.87909 |
| Itgb1 | integrin beta 1 (fibronectin receptor beta) [Source:MGI Symbol;Acc:MGI:96610] | axon development, tissue migration, cell-substrate adhesion, cell leading edge, extracellular matrix binding | -1.27078 | 1.55091 |
| Jmy | junction-mediating and regulatory protein [Source:MGI Symbol;Acc:MGI:1913096] | regulation of supramolecular fiber organization, cell leading edge | -0.813646 |  |
| Jun | jun proto-oncogene [Source:MGI Symbol;Acc:MGI:96646] | axon development, tissue migration |  | 1.09278 |
| Jup | junction plakoglobin [Source:MGI Symbol;Acc:MGI:96650] | tissue migration, cell-substrate adhesion |  | -1.30925 |
| Kank1 | KN motif and ankyrin repeat domains 1 [Source:MGI Symbol;Acc:MGI:2147707] | regulation of supramolecular fiber organization, tissue migration, cell-substrate adhesion, cell leading edge |  | 2.80551 |
| Kank2 | KN motif and ankyrin repeat domains 2 [Source:MGI Symbol;Acc:MGI:2384568] | regulation of supramolecular fiber organization, tissue migration |  | 2.72834 |
| Kcna6 | potassium voltage-gated channel, shaker-related, subfamily, member 6 [Source:MGI Symbol;Acc:MGI:96663] | axon part |  | -1.81428 |
| Kcnb1 | potassium voltage gated channel, Shab-related subfamily, member 1 [Source:MGI Symbol;Acc:MGI:96666] | cell leading edge |  | -1.57546 |
| Kcnc1 | potassium voltage gated channel, Shaw-related subfamily, member 1 [Source:MGI Symbol;Acc:MGI:96667] | axon part, cell leading edge |  | -1.01559 |
| Kcnc2 | potassium voltage gated channel, Shaw-related subfamily, member 2 [Source:MGI Symbol;Acc:MGI:96668] | axon part, cell leading edge |  | -2.31674 |
| Kcnip3 | Kv channel interacting protein 3, calsenilin [Source:MGI Symbol;Acc:MGI:1929258] | axon part |  | 3.44892 |
| Kcnq2 | potassium voltage-gated channel, subfamily Q, member 2 [Source:MGI Symbol;Acc:MGI:1309503] | axon part |  | -1.48528 |
| Kif1b | kinesin family member 1B [Source:MGI Symbol;Acc:MGI:108426] | axon part | -0.626212 | -0.902574 |
| Kif3a | kinesin family member 3A [Source:MGI Symbol;Acc:MGI:107689] | axon development, axon part | -1.29656 | -1.54262 |
| Klhl2 | kelch-like 2, Mayven [Source:MGI Symbol;Acc:MGI:1924363] | cell leading edge |  | -1.43319 |
| Lamb1 | laminin B1 [Source:MGI Symbol;Acc:MGI:96743] | cell-substrate adhesion |  | 4.4541 |
| Ldb2 | LIM domain binding 2 [Source:MGI Symbol;Acc:MGI:894670] | cell leading edge | 1.0571 |  |
| Lef1 | lymphoid enhancer binding factor 1 [Source:MGI Symbol;Acc:MGI:96770] | morphogenesis of a branching structure | 1.97895 |  |
| Lgi1 | leucine-rich repeat LGI family, member 1 [Source:MGI Symbol;Acc:MGI:1861691] | axon development |  | -1.42152 |
| Lgmn | legumain [Source:MGI Symbol;Acc:MGI:1330838] | tissue migration |  | 3.43697 |
| Lima1 | LIM domain and actin binding 1 [Source:MGI Symbol;Acc:MGI:1920992] | regulation of supramolecular fiber organization, cell leading edge |  | 2.40127 |
| Lmtk2 | lemur tyrosine kinase 2 [Source:MGI Symbol;Acc:MGI:3036247] | axon development, axon part | -0.628425 | -2.07108 |
| Lpar1 | lysophosphatidic acid receptor 1 [Source:MGI Symbol;Acc:MGI:108429] | regulation of supramolecular fiber organization | -1.64849 |  |
| Lrig2 | leucine-rich repeats and immunoglobulin-like domains 2 [Source:MGI Symbol;Acc:MGI:2443718] | axon development, axon part |  | -1.33965 |
| Lrp4 | low density lipoprotein receptor-related protein 4 [Source:MGI Symbol;Acc:MGI:2442252] | axon development |  | 2.77733 |
| Lrrc4c | leucine rich repeat containing 4C [Source:MGI Symbol;Acc:MGI:2442636] | axon development | -0.705496 |  |
| Magel2 | melanoma antigen, family L, 2 [Source:MGI Symbol;Acc:MGI:1351648] | regulation of supramolecular fiber organization |  | -2.89638 |
| Map1a | microtubule-associated protein 1 A [Source:MGI Symbol;Acc:MGI:1306776] | axon development, regulation of supramolecular fiber organization, axon part | -0.666585 |  |
| Map2 | microtubule-associated protein 2 [Source:MGI Symbol;Acc:MGI:97175] | axon development, regulation of supramolecular fiber organization, axon part, cell leading edge |  | 0.816556 |
| Map4k4 | mitogen-activated protein kinase kinase kinase kinase 4 [Source:MGI Symbol;Acc:MGI:1349394] | tissue migration, cell-substrate adhesion | -0.602763 |  |
| Map6 | microtubule-associated protein 6 [Source:MGI Symbol;Acc:MGI:1201690] | axon development, axon part |  | -1.75568 |
| Mapre1 | microtubule-associated protein, RP/EB family, member 1 [Source:MGI Symbol;Acc:MGI:891995] | regulation of supramolecular fiber organization | -0.686779 | -1.24738 |
| Mapre2 | microtubule-associated protein, RP/EB family, member 2 [Source:MGI Symbol;Acc:MGI:106271] | tissue migration |  | -2.00285 |
| Mapt | microtubule-associated protein tau [Source:MGI Symbol;Acc:MGI:97180] | axon development, regulation of supramolecular fiber organization, axon part, cell leading edge |  | -1.67853 |
| Mcc | mutated in colorectal cancers [Source:MGI Symbol;Acc:MGI:96930] | tissue migration | -0.795106 |  |
| Mecp2 | methyl CpG binding protein 2 [Source:MGI Symbol;Acc:MGI:99918] | regulation of supramolecular fiber organization, tissue migration, morphogenesis of a branching structure | -0.614402 | -1.18061 |
| Mef2c | myocyte enhancer factor 2C [Source:MGI Symbol;Acc:MGI:99458] | regulation of supramolecular fiber organization, tissue migration |  | 2.4051 |
| Men1 | multiple endocrine neoplasia 1 [Source:MGI Symbol;Acc:MGI:1316736] | cell-substrate adhesion | 1.20735 |  |
| Metrn | meteorin, glial cell differentiation regulator [Source:MGI Symbol;Acc:MGI:1917333] | axon development |  | 3.42335 |
| Mia3 | melanoma inhibitory activity 3 [Source:MGI Symbol;Acc:MGI:2443183] | tissue migration | -0.828284 |  |
| Mmp14 | matrix metallopeptidase 14 (membrane-inserted) [Source:MGI Symbol;Acc:MGI:101900] | cell-substrate adhesion, morphogenesis of a branching structure |  | 3.89168 |
| Mmp9 | matrix metallopeptidase 9 [Source:MGI Symbol;Acc:MGI:97011] | tissue migration |  | 3.51627 |
| Mpp2 | membrane protein, palmitoylated 2 (MAGUK p55 subfamily member 2) [Source:MGI Symbol;Acc:MGI:1858257] | cell leading edge |  | -2.72701 |
| Mtr | 5-methyltetrahydrofolate-homocysteine methyltransferase [Source:MGI Symbol;Acc:MGI:894292] | axon development |  | 2.46132 |
| Mtss1 | metastasis suppressor 1 [Source:MGI Symbol;Acc:MGI:2384818] | regulation of supramolecular fiber organization |  | -1.42134 |
| Myh9 | myosin, heavy polypeptide 9, non-muscle [Source:MGI Symbol;Acc:MGI:107717] | regulation of supramolecular fiber organization, tissue migration, cell leading edge |  | 3.78983 |
| Myo10 | myosin X [Source:MGI Symbol;Acc:MGI:107716] | cell leading edge |  | 2.42607 |
| Nanos1 | nanos C2HC-type zinc finger 1 [Source:MGI Symbol;Acc:MGI:2669254] | tissue migration |  | -1.69318 |
| Nav1 | neuron navigator 1 [Source:MGI Symbol;Acc:MGI:2183683] | axon part |  | -1.52079 |
| Nav3 | neuron navigator 3 [Source:MGI Symbol;Acc:MGI:2183703] | regulation of supramolecular fiber organization |  | -1.54438 |
| Ncam1 | neural cell adhesion molecule 1 [Source:MGI Symbol;Acc:MGI:97281] | axon development, neuron projection fasciculation, axon part |  | -1.25731 |
| Ndn | necdin [Source:MGI Symbol;Acc:MGI:97290] | axon development, neuron projection fasciculation | -0.632285 | -0.884451 |
| Ndnf | neuron-derived neurotrophic factor [Source:MGI Symbol;Acc:MGI:1915419] | cell-substrate adhesion |  | -2.11619 |
| Ndrg2 | N-myc downstream regulated gene 2 [Source:MGI Symbol;Acc:MGI:1352498] | axon part |  | 2.50966 |
| Nfasc | neurofascin [Source:MGI Symbol;Acc:MGI:104753] | axon development, axon part |  | -1.47021 |
| Nfe2l2 | nuclear factor, erythroid derived 2, like 2 [Source:MGI Symbol;Acc:MGI:108420] | tissue migration |  | 5.005 |
| Nid1 | nidogen 1 [Source:MGI Symbol;Acc:MGI:97342] | cell-substrate adhesion, extracellular matrix binding |  | 2.71017 |
| Nkx6-1 | NK6 homeobox 1 [Source:MGI Symbol;Acc:MGI:1206039] | axon development |  | -2.17344 |
| Nme1 | NME/NM23 nucleoside diphosphate kinase 1 [Source:MGI Symbol;Acc:MGI:97355] | cell leading edge |  | -1.34415 |
| Notch1 | notch 1 [Source:MGI Symbol;Acc:MGI:97363] | axon development, tissue migration, cell-substrate adhesion, morphogenesis of a branching structure, cell leading edge |  | 1.45405 |
| Nptx1 | neuronal pentraxin 1 [Source:MGI Symbol;Acc:MGI:107811] | axon development |  | -1.97758 |
| Npy | neuropeptide Y [Source:MGI Symbol;Acc:MGI:97374] | axon part | 2.61346 |  |
| Npy2r | neuropeptide Y receptor Y2 [Source:MGI Symbol;Acc:MGI:108418] | cell-substrate adhesion | -2.49744 | -2.89667 |
| **Nr2f2** | **nuclear receptor subfamily 2, group F, member 2 [Source:MGI Symbol;Acc:MGI:1352452]** | **tissue migration** | **-0.733782** | **-2.56305** |
| Nrbp1 | nuclear receptor binding protein 1 [Source:MGI Symbol;Acc:MGI:2183436] | cell leading edge | -0.609058 |  |
| Nrg3 | neuregulin 3 [Source:MGI Symbol;Acc:MGI:1097165] | negative chemotaxis |  | -1.53498 |
| Nrp2 | neuropilin 2 [Source:MGI Symbol;Acc:MGI:1100492] | axon development, negative chemotaxis | -0.982098 |  |
| Nrsn1 | neurensin 1 [Source:MGI Symbol;Acc:MGI:894662] | axon part |  | -2.14189 |
| Ntn1 | netrin 1 [Source:MGI Symbol;Acc:MGI:105088] | axon development |  | 2.69235 |
| Ntng1 | netrin G1 [Source:MGI Symbol;Acc:MGI:1934028] | axon development |  | -2.32904 |
| Ntrk2 | neurotrophic tyrosine kinase, receptor, type 2 [Source:MGI Symbol;Acc:MGI:97384] | axon development, axon part | -0.637499 |  |
| Numbl | numb-like [Source:MGI Symbol;Acc:MGI:894702] | axon development |  | -2.03233 |
| Olfm1 | olfactomedin 1 [Source:MGI Symbol;Acc:MGI:1860437] | axon development, axon part |  | -1.1814 |
| Oprk1 | opioid receptor, kappa 1 [Source:MGI Symbol;Acc:MGI:97439] | axon part |  | -2.89531 |
| Orai2 | ORAI calcium release-activated calcium modulator 2 [Source:MGI Symbol;Acc:MGI:2443195] | axon part |  | -1.48563 |
| Pacsin2 | protein kinase C and casein kinase substrate in neurons 2 [Source:MGI Symbol;Acc:MGI:1345153] | cell leading edge |  | 1.74718 |
| Pak3 | p21 (RAC1) activated kinase 3 [Source:MGI Symbol;Acc:MGI:1339656] | axon development, regulation of supramolecular fiber organization |  | -1.01958 |
| Pax2 | paired box 2 [Source:MGI Symbol;Acc:MGI:97486] | morphogenesis of a branching structure | 2.31743 |  |
| Pax8 | paired box 8 [Source:MGI Symbol;Acc:MGI:97492] | morphogenesis of a branching structure |  | 3.35012 |
| Paxip1 | PAX interacting (with transcription-activation domain) protein 1 [Source:MGI Symbol;Acc:MGI:1890430] | tissue migration |  | -1.19749 |
| **Pcdhac2** | **protocadherin alpha subfamily C, 2 [Source:MGI Symbol;Acc:MGI:1891443]** | **cell adhesion** | **-1.82227** | **-3.6608** |
| **Pcsk5** | **proprotein convertase subtilisin/kexin type 5 [Source:MGI Symbol;Acc:MGI:97515]** | **cell-substrate adhesion** | **-2.27439** | **-3.43747** |
| Pdgfra | platelet derived growth factor receptor, alpha polypeptide [Source:MGI Symbol;Acc:MGI:97530] | morphogenesis of a branching structure |  | 2.68538 |
| Pdpn | podoplanin [Source:MGI Symbol;Acc:MGI:103098] | cell-substrate adhesion, cell leading edge |  | 3.65016 |
| Pdxp | pyridoxal (pyridoxine, vitamin B6) phosphatase [Source:MGI Symbol;Acc:MGI:1919282] | regulation of supramolecular fiber organization, cell leading edge |  | -2.02707 |
| Penk | preproenkephalin [Source:MGI Symbol;Acc:MGI:104629] | axon part | -1.31513 | -2.58404 |
| Pgr | progesterone receptor [Source:MGI Symbol;Acc:MGI:97567] | morphogenesis of a branching structure, axon part | -1.92335 | -2.48984 |
| Picalm | phosphatidylinositol binding clathrin assembly protein [Source:MGI Symbol;Acc:MGI:2385902] | axon development | -0.647028 |  |
| Pitpna | phosphatidylinositol transfer protein, alpha [Source:MGI Symbol;Acc:MGI:99887] | axon development | -0.599331 |  |
| Pkd2 | polycystic kidney disease 2 [Source:MGI Symbol;Acc:MGI:1099818] | morphogenesis of a branching structure, cell leading edge |  | 1.62692 |
| Plek | pleckstrin [Source:MGI Symbol;Acc:MGI:1860485] | regulation of supramolecular fiber organization, cell leading edge |  | 3.88851 |
| Plekhg2 | pleckstrin homology domain containing, family G (with RhoGef domain) member 2 [Source:MGI Symbol;Acc:MGI:2141874] | regulation of supramolecular fiber organization |  | 2.37471 |
| Plxna1 | plexin A1 [Source:MGI Symbol;Acc:MGI:107685] | axon development, morphogenesis of a branching structure |  | -1.40299 |
| Plxna3 | plexin A3 [Source:MGI Symbol;Acc:MGI:107683] | axon development, negative chemotaxis |  | -1.32445 |
| Plxna4 | plexin A4 [Source:MGI Symbol;Acc:MGI:2179061] | axon development, negative chemotaxis |  | -0.925581 |
| Plxnb2 | plexin B2 [Source:MGI Symbol;Acc:MGI:2154239] | axon development |  | 0.952712 |
| Plxnc1 | plexin C1 [Source:MGI Symbol;Acc:MGI:1890127] | axon development |  | -1.25938 |
| Pmp22 | peripheral myelin protein 22 [Source:MGI Symbol;Acc:MGI:97631] | axon development |  | 4.45394 |
| Ppard | peroxisome proliferator activator receptor delta [Source:MGI Symbol;Acc:MGI:101884] | tissue migration, cell-substrate adhesion |  | 2.3985 |
| Ppp3r1 | protein phosphatase 3, regulatory subunit B, alpha isoform (calcineurin B, type I) [Source:MGI Symbol;Acc:MGI:107172] | morphogenesis of a branching structure | -0.654313 | -1.34912 |
| Prex1 | phosphatidylinositol-3,4,5-trisphosphate-dependent Rac exchange factor 1 [Source:MGI Symbol;Acc:MGI:3040696] | regulation of supramolecular fiber organization, cell-substrate adhesion, axon part |  | 2.66111 |
| Prkcb | protein kinase C, beta [Source:MGI Symbol;Acc:MGI:97596] | axon part |  | -0.776683 |
| Prkce | protein kinase C, epsilon [Source:MGI Symbol;Acc:MGI:97599] | regulation of supramolecular fiber organization, tissue migration, cell-substrate adhesion |  | -1.1059 |
| Prkcq | protein kinase C, theta [Source:MGI Symbol;Acc:MGI:97601] | regulation of supramolecular fiber organization | -1.29833 |  |
| Prkcz | protein kinase C, zeta [Source:MGI Symbol;Acc:MGI:97602] | cell-substrate adhesion, axon part, cell leading edge |  | -1.7815 |
| Prrt2 | proline-rich transmembrane protein 2 [Source:MGI Symbol;Acc:MGI:1916267] | axon part |  | -1.27291 |
| Psd | pleckstrin and Sec7 domain containing [Source:MGI Symbol;Acc:MGI:1920978] | cell leading edge |  | -2.21259 |
| Psd3 | pleckstrin and Sec7 domain containing 3 [Source:MGI Symbol;Acc:MGI:1918215] | cell leading edge |  | -1.05814 |
| Ptbp2 | polypyrimidine tract binding protein 2 [Source:MGI Symbol;Acc:MGI:1860489] | axon part |  | -1.03372 |
| Ptger4 | prostaglandin E receptor 4 (subtype EP4) [Source:MGI Symbol;Acc:MGI:104311] | regulation of supramolecular fiber organization |  | -3.81965 |
| Ptn | pleiotrophin [Source:MGI Symbol;Acc:MGI:97804] | cell-substrate adhesion |  | 2.46984 |
| Ptpn13 | protein tyrosine phosphatase, non-receptor type 13 [Source:MGI Symbol;Acc:MGI:103293] | cell leading edge |  | 1.72154 |
| Ptprf | protein tyrosine phosphatase, receptor type, F [Source:MGI Symbol;Acc:MGI:102695] | axon development, axon part | -0.63967 |  |
| Ptprm | protein tyrosine phosphatase, receptor type, M [Source:MGI Symbol;Acc:MGI:102694] | axon development, tissue migration, cell leading edge | -0.774414 | -1.06726 |
| Ptprn2 | protein tyrosine phosphatase, receptor type, N polypeptide 2 [Source:MGI Symbol;Acc:MGI:107418] | axon part |  | -1.36907 |
| Ptpro | protein tyrosine phosphatase, receptor type, O [Source:MGI Symbol;Acc:MGI:1097152] | axon development |  | -1.49485 |
| Ptprr | protein tyrosine phosphatase, receptor type, R [Source:MGI Symbol;Acc:MGI:109559] | tissue migration |  | -1.5954 |
| Ptprs | protein tyrosine phosphatase, receptor type, S [Source:MGI Symbol;Acc:MGI:97815] | axon development |  | -1.47617 |
| Ptprz1 | protein tyrosine phosphatase, receptor type Z, polypeptide 1 [Source:MGI Symbol;Acc:MGI:97816] | axon development, neuron projection fasciculation, cell-substrate adhesion, axon part, cell leading edge |  | 1.60443 |
| Rab10 | RAB10, member RAS oncogene family [Source:MGI Symbol;Acc:MGI:105066] | axon development | -0.866468 | -0.802575 |
| Rab3a | RAB3A, member RAS oncogene family [Source:MGI Symbol;Acc:MGI:97843] | axon development, axon part |  | -2.01307 |
| Rac2 | Rac family small GTPase 2 [Source:MGI Symbol;Acc:MGI:97846] | cell-substrate adhesion, cell leading edge |  | 4.68948 |
| Rac3 | Rac family small GTPase 3 [Source:MGI Symbol;Acc:MGI:2180784] | regulation of supramolecular fiber organization, cell-substrate adhesion, axon part, cell leading edge |  | -1.57276 |
| **Rangap1** | **RAN GTPase activating protein 1 [Source:MGI Symbol;Acc:MGI:103071]** | **axon part** | **-1.11683** | **-1.85507** |
| Raph1 | Ras association (RalGDS/AF-6) and pleckstrin homology domains 1 [Source:MGI Symbol;Acc:MGI:1924550] | axon development, cell leading edge | -0.734261 | -1.08447 |
| Rasgrf1 | RAS protein-specific guanine nucleotide-releasing factor 1 [Source:MGI Symbol;Acc:MGI:99694] | axon part |  | -2.65424 |
| Rdx | radixin [Source:MGI Symbol;Acc:MGI:97887] | regulation of supramolecular fiber organization, cell leading edge |  | 2.20706 |
| Reln | reelin [Source:MGI Symbol;Acc:MGI:103022] | axon development |  | -2.58788 |
| Ret | ret proto-oncogene [Source:MGI Symbol;Acc:MGI:97902] | axon development | -0.92342 | -2.58715 |
| Rgcc | regulator of cell cycle [Source:MGI Symbol;Acc:MGI:1913464] | regulation of supramolecular fiber organization, tissue migration |  | 4.87577 |
| Rgma | repulsive guidance molecule family member A [Source:MGI Symbol;Acc:MGI:2679262] | axon development | 0.74546 |  |
| Rgs10 | regulator of G-protein signalling 10 [Source:MGI Symbol;Acc:MGI:1915115] | axon part | 1.07879 |  |
| Rhoa | ras homolog family member A [Source:MGI Symbol;Acc:MGI:1096342] | regulation of supramolecular fiber organization, tissue migration, cell-substrate adhesion, negative chemotaxis, cell leading edge | -0.796751 |  |
| **Rhob** | **ras homolog family member B [Source:MGI Symbol;Acc:MGI:107949]** | **tissue migration** | **-0.628854** | **-0.97183** |
| Rhoj | ras homolog family member J [Source:MGI Symbol;Acc:MGI:1931551] | tissue migration |  | 3.26961 |
| Rit2 | Ras-like without CAAX 2 [Source:MGI Symbol;Acc:MGI:108054] | semaphorin receptor binding |  | -2.25335 |
| Rps3 | ribosomal protein S3 [Source:MGI Symbol;Acc:MGI:1350917] | regulation of supramolecular fiber organization, cell leading edge |  | -1.66868 |
| Rreb1 | ras responsive element binding protein 1 [Source:MGI Symbol;Acc:MGI:2443664] | tissue migration, cell-substrate adhesion |  | 4.05549 |
| Rtn4 | reticulon 4 [Source:MGI Symbol;Acc:MGI:1915835] | axon development, tissue migration, neuron projection fasciculation, morphogenesis of a branching structure | -0.752739 | -0.782122 |
| Rtn4rl1 | reticulon 4 receptor-like 1 [Source:MGI Symbol;Acc:MGI:2661375] | axon development |  | 2.02618 |
| Rufy3 | RUN and FYVE domain containing 3 [Source:MGI Symbol;Acc:MGI:106484] | axon development, axon part, cell leading edge | -0.854967 | -0.981065 |
| Ryk | receptor-like tyrosine kinase [Source:MGI Symbol;Acc:MGI:101766] | axon development, negative chemotaxis | -0.846894 |  |
| S100a10 | S100 calcium binding protein A10 (calpactin) [Source:MGI Symbol;Acc:MGI:1339468] | regulation of supramolecular fiber organization, cell-substrate adhesion | -1.73933 | -4.08309 |
| S100a11 | S100 calcium binding protein A11 [Source:MGI Symbol;Acc:MGI:1338798] | cell leading edge | -0.753519 |  |
| S100b | S100 protein, beta polypeptide, neural [Source:MGI Symbol;Acc:MGI:98217] | cell leading edge |  | 3.11031 |
| S1pr1 | sphingosine-1-phosphate receptor 1 [Source:MGI Symbol;Acc:MGI:1096355] | regulation of supramolecular fiber organization |  | 3.77763 |
| Scarb1 | scavenger receptor class B, member 1 [Source:MGI Symbol;Acc:MGI:893578] | tissue migration | 0.908871 |  |
| Scn8a | sodium channel, voltage-gated, type VIII, alpha [Source:MGI Symbol;Acc:MGI:103169] | axon part | -0.73226 |  |
| Sema3c | sema domain, immunoglobulin domain (Ig), short basic domain, secreted, (semaphorin) 3C [Source:MGI Symbol;Acc:MGI:107557] | axon development, negative chemotaxis, morphogenesis of a branching structure, semaphorin receptor binding | -1.56475 |  |
| Sema4a | sema domain, immunoglobulin domain (Ig), transmembrane domain (TM) and short cytoplasmic domain, (semaphorin) 4A [Source:MGI Symbol;Acc:MGI:107560] | axon development, tissue migration, negative chemotaxis, semaphorin receptor binding |  | -1.68114 |
| Sema4d | sema domain, immunoglobulin domain (Ig), transmembrane domain (TM) and short cytoplasmic domain, (semaphorin) 4D [Source:MGI Symbol;Acc:MGI:109244] | axon development, negative chemotaxis, semaphorin receptor binding | -0.753722 |  |
| Sema4f | sema domain, immunoglobulin domain (Ig), TM domain, and short cytoplasmic domain [Source:MGI Symbol;Acc:MGI:1340055] | axon development, negative chemotaxis, semaphorin receptor binding |  | -1.79924 |
| Sema4g | sema domain, immunoglobulin domain (Ig), transmembrane domain (TM) and short cytoplasmic domain, (semaphorin) 4G [Source:MGI Symbol;Acc:MGI:1347047] | axon development, negative chemotaxis, semaphorin receptor binding |  | -1.82732 |
| Sema5b | sema domain, seven thrombospondin repeats (type 1 and type 1-like), transmembrane domain (TM) and short cytoplasmic domain, (semaphorin) 5B [Source:MGI Symbol;Acc:MGI:107555] | axon development, negative chemotaxis, semaphorin receptor binding |  | 1.99165 |
| Sfrp1 | secreted frizzled-related protein 1 [Source:MGI Symbol;Acc:MGI:892014] | cell-substrate adhesion, morphogenesis of a branching structure |  | 2.45135 |
| Sfrp2 | secreted frizzled-related protein 2 [Source:MGI Symbol;Acc:MGI:108078] | morphogenesis of a branching structure |  | 2.18826 |
| Sgk1 | serum/glucocorticoid regulated kinase 1 [Source:MGI Symbol;Acc:MGI:1340062] | regulation of supramolecular fiber organization | -0.873794 |  |
| Sh3pxd2b | SH3 and PX domains 2B [Source:MGI Symbol;Acc:MGI:2442062] | regulation of supramolecular fiber organization |  | -1.46685 |
| Sh3rf1 | SH3 domain containing ring finger 1 [Source:MGI Symbol;Acc:MGI:1913066] | cell leading edge |  | -2.23312 |
| Shank2 | SH3 and multiple ankyrin repeat domains 2 [Source:MGI Symbol;Acc:MGI:2671987] | axon part | 1.02854 |  |
| Shank3 | SH3 and multiple ankyrin repeat domains 3 [Source:MGI Symbol;Acc:MGI:1930016] | regulation of supramolecular fiber organization |  | -2.17284 |
| Shisa7 | shisa family member 7 [Source:MGI Symbol;Acc:MGI:3605641] | cell leading edge |  | -1.8682 |
| Shox2 | short stature homeobox 2 [Source:MGI Symbol;Acc:MGI:1201673] | axon development, morphogenesis of a branching structure |  | -1.72665 |
| Sirpa | signal-regulatory protein alpha [Source:MGI Symbol;Acc:MGI:108563] | cell-substrate adhesion |  | 1.6794 |
| Sirt1 | sirtuin 1 [Source:MGI Symbol;Acc:MGI:2135607] | tissue migration, axon part |  | -1.9342 |
| Slc12a2 | solute carrier family 12, member 2 [Source:MGI Symbol;Acc:MGI:101924] | morphogenesis of a branching structure |  | 2.106 |
| Slc12a5 | solute carrier family 12, member 5 [Source:MGI Symbol;Acc:MGI:1862037] | cell leading edge |  | -1.52563 |
| **Slc17a6** | **solute carrier family 17 (sodium-dependent inorganic phosphate cotransporter), member 6 [Source:MGI Symbol;Acc:MGI:2156052]** | **axon part** | **1.37734** |  |
| Slc17a8 | solute carrier family 17 (sodium-dependent inorganic phosphate cotransporter), member 8 [Source:MGI Symbol;Acc:MGI:3039629] | axon part |  | -3.06482 |
| **Slc18a2** | **solute carrier family 18 (vesicular monoamine), member 2 [Source:MGI Symbol;Acc:MGI:106677]** | **axon part** | **-2.40196** | **-3.97069** |
| Slc9a3r1 | solute carrier family 9 (sodium/hydrogen exchanger), member 3 regulator 1 [Source:MGI Symbol;Acc:MGI:1349482] | cell leading edge |  | 2.59146 |
| Slc9a6 | solute carrier family 9 (sodium/hydrogen exchanger), member 6 [Source:MGI Symbol;Acc:MGI:2443511] | axon development, axon part |  | -1.32038 |
| Slit2 | slit guidance ligand 2 [Source:MGI Symbol;Acc:MGI:1315205] | axon development, regulation of supramolecular fiber organization, tissue migration, negative chemotaxis, morphogenesis of a branching structure, extracellular matrix binding | -1.40234 |  |
| Slitrk1 | SLIT and NTRK-like family, member 1 [Source:MGI Symbol;Acc:MGI:2679446] | axon development |  | -2.5382 |
| Slitrk3 | SLIT and NTRK-like family, member 3 [Source:MGI Symbol;Acc:MGI:2679447] | axon development |  | -1.32093 |
| Slitrk4 | SLIT and NTRK-like family, member 4 [Source:MGI Symbol;Acc:MGI:2442509] | axon development |  | -2.0305 |
| Slitrk5 | SLIT and NTRK-like family, member 5 [Source:MGI Symbol;Acc:MGI:2679448] | axon development | -0.838027 | -1.61212 |
| Smo | smoothened, frizzled class receptor [Source:MGI Symbol;Acc:MGI:108075] | axon development, morphogenesis of a branching structure, axon part |  | 3.19804 |
| Smoc1 | SPARC related modular calcium binding 1 [Source:MGI Symbol;Acc:MGI:1929878] | cell-substrate adhesion, extracellular matrix binding |  | 2.84505 |
| Snap91 | synaptosomal-associated protein 91 [Source:MGI Symbol;Acc:MGI:109132] | axon development, axon part |  | -1.27 |
| Sncb | synuclein, beta [Source:MGI Symbol;Acc:MGI:1889011] | axon part |  | -2.27091 |
| Sncg | synuclein, gamma [Source:MGI Symbol;Acc:MGI:1298397] | axon part |  | -2.29134 |
| Snx5 | sorting nexin 5 [Source:MGI Symbol;Acc:MGI:1916428] | cell leading edge |  | 2.55875 |
| Snx9 | sorting nexin 9 [Source:MGI Symbol;Acc:MGI:1913866] | cell leading edge |  | 2.25576 |
| Sox9 | SRY (sex determining region Y)-box 9 [Source:MGI Symbol;Acc:MGI:98371] | tissue migration, morphogenesis of a branching structure |  | 1.89538 |
| Sp1 | trans-acting transcription factor 1 [Source:MGI Symbol;Acc:MGI:98372] | tissue migration | -0.861824 |  |
| Sparc | secreted acidic cysteine rich glycoprotein [Source:MGI Symbol;Acc:MGI:98373] | tissue migration, extracellular matrix binding |  | 4.73567 |
| Sparcl1 | SPARC-like 1 [Source:MGI Symbol;Acc:MGI:108110] | extracellular matrix binding |  | 2.96139 |
| Spata13 | spermatogenesis associated 13 [Source:MGI Symbol;Acc:MGI:104838] | cell leading edge |  | 2.86461 |
| Spock2 | sparc/osteonectin, cwcv and kazal-like domains proteoglycan 2 [Source:MGI Symbol;Acc:MGI:1891351] | cell-substrate adhesion, extracellular matrix binding |  | -1.58152 |
| Spock3 | sparc/osteonectin, cwcv and kazal-like domains proteoglycan 3 [Source:MGI Symbol;Acc:MGI:1920152] | extracellular matrix binding | -0.783579 | -1.76355 |
| Spry2 | sprouty RTK signaling antagonist 2 [Source:MGI Symbol;Acc:MGI:1345138] | morphogenesis of a branching structure, cell leading edge |  | 2.30277 |
| Sptbn1 | spectrin beta, non-erythrocytic 1 [Source:MGI Symbol;Acc:MGI:98388] | regulation of supramolecular fiber organization, axon part, cell leading edge | -0.627532 |  |
| Srcin1 | SRC kinase signaling inhibitor 1 [Source:MGI Symbol;Acc:MGI:1933179] | cell-substrate adhesion, cell leading edge | -0.999121 | -1.3632 |
| Ssh2 | slingshot protein phosphatase 2 [Source:MGI Symbol;Acc:MGI:2679255] | axon development, regulation of supramolecular fiber organization | -0.671567 |  |
| Stard13 | StAR-related lipid transfer (START) domain containing 13 [Source:MGI Symbol;Acc:MGI:2385331] | tissue migration | -1.82446 | -1.89672 |
| Stk25 | serine/threonine kinase 25 (yeast) [Source:MGI Symbol;Acc:MGI:1891699] | axon development |  | -1.13159 |
| Stmn2 | stathmin-like 2 [Source:MGI Symbol;Acc:MGI:98241] | regulation of supramolecular fiber organization, axon part, cell leading edge | -0.640204 | -1.04456 |
| Stmn3 | stathmin-like 3 [Source:MGI Symbol;Acc:MGI:1277137] | axon part |  | -2.26573 |
| Stmn4 | stathmin-like 4 [Source:MGI Symbol;Acc:MGI:1931224] | axon part | -0.593573 | -2.01138 |
| Stx3 | syntaxin 3 [Source:MGI Symbol;Acc:MGI:103077] | axon part, cell leading edge | -1.10137 |  |
| Stxbp1 | syntaxin binding protein 1 [Source:MGI Symbol;Acc:MGI:107363] | axon development |  | -1.86731 |
| Stxbp5 | syntaxin binding protein 5 (tomosyn) [Source:MGI Symbol;Acc:MGI:1926058] | axon development | -0.703511 | -1.01002 |
| Sulf1 | sulfatase 1 [Source:MGI Symbol;Acc:MGI:2138563] | morphogenesis of a branching structure |  | 1.88848 |
| **Syp** | **synaptophysin [Source:MGI Symbol;Acc:MGI:98467]** | **axon part** | **-0.776693** | **-2.5161** |
| Syt11 | synaptotagmin XI [Source:MGI Symbol;Acc:MGI:1859547] | axon part | -0.806483 |  |
| Syt7 | synaptotagmin VII [Source:MGI Symbol;Acc:MGI:1859545] | axon part |  | -1.54504 |
| Tac1 | tachykinin 1 [Source:MGI Symbol;Acc:MGI:98474] | regulation of supramolecular fiber organization, tissue migration | 1.67824 | -4.59322 |
| Tacr3 | tachykinin receptor 3 [Source:MGI Symbol;Acc:MGI:892968] | cell leading edge | -1.41873 |  |
| Tbc1d24 | TBC1 domain family, member 24 [Source:MGI Symbol;Acc:MGI:2443456] | axon part | -0.665784 |  |
| Tgfbr1 | transforming growth factor, beta receptor I [Source:MGI Symbol;Acc:MGI:98728] | regulation of supramolecular fiber organization, tissue migration | -1.12567 | 1.61954 |
| Them4 | thioesterase superfamily member 4 [Source:MGI Symbol;Acc:MGI:1923028] | cell leading edge |  | -1.6154 |
| Timeless | timeless circadian clock 1 [Source:MGI Symbol;Acc:MGI:1321393] | morphogenesis of a branching structure |  | 4.0778 |
| Tln1 | talin 1 [Source:MGI Symbol;Acc:MGI:1099832] | cell leading edge |  | 2.78874 |
| Tlr2 | toll-like receptor 2 [Source:MGI Symbol;Acc:MGI:1346060] | regulation of supramolecular fiber organization |  | 3.66994 |
| Tmeff2 | transmembrane protein with EGF-like and two follistatin-like domains 2 [Source:MGI Symbol;Acc:MGI:1861735] | regulation of supramolecular fiber organization | -1.34819 | -1.18733 |
| Tmem8b | transmembrane protein 8B [Source:MGI Symbol;Acc:MGI:2441680] | cell-substrate adhesion |  | -2.37205 |
| Tmod2 | tropomodulin 2 [Source:MGI Symbol;Acc:MGI:1355335] | regulation of supramolecular fiber organization, axon part | -0.627287 | -0.957407 |
| Tmsb10 | thymosin, beta 10 [Source:MGI Symbol;Acc:MGI:109146] | regulation of supramolecular fiber organization |  | -1.68187 |
| **Tnc** | **tenascin C [Source:MGI Symbol;Acc:MGI:101922]** | **axon development, morphogenesis of a branching structure** | **1.2064** | **2.77012** |
| **Tnfrsf21** | **tumor necrosis factor receptor superfamily, member 21 [Source:MGI Symbol;Acc:MGI:2151075]** | **axon development, neuron projection fasciculation** | **-0.612938** | **-0.874129** |
| Tpm3 | tropomyosin 3, gamma [Source:MGI Symbol;Acc:MGI:1890149] | axon part | -0.881865 |  |
| Tpx2 | TPX2, microtubule-associated [Source:MGI Symbol;Acc:MGI:1919369] | axon part |  | 1.99256 |
| Trak2 | trafficking protein, kinesin binding 2 [Source:MGI Symbol;Acc:MGI:1918077] | axon development, axon part | -0.792756 |  |
| Trim46 | tripartite motif-containing 46 [Source:MGI Symbol;Acc:MGI:2673000] | axon development, axon part |  | -2.4763 |
| Trpv2 | transient receptor potential cation channel, subfamily V, member 2 [Source:MGI Symbol;Acc:MGI:1341836] | axon development, axon part, cell leading edge |  | -1.91614 |
| Tshz3 | teashirt zinc finger family member 3 [Source:MGI Symbol;Acc:MGI:2442819] | axon part |  | -1.58469 |
| Ttyh1 | tweety family member 1 [Source:MGI Symbol;Acc:MGI:1889007] | cell-substrate adhesion |  | 2.56986 |
| Tubb3 | tubulin, beta 3 class III [Source:MGI Symbol;Acc:MGI:107813] | axon development |  | -1.99367 |
| Tubb4a | tubulin, beta 4A class IVA [Source:MGI Symbol;Acc:MGI:107848] | regulation of supramolecular fiber organization, axon part |  | -1.01426 |
| Twf1 | twinfilin actin binding protein 1 [Source:MGI Symbol;Acc:MGI:1100520] | regulation of supramolecular fiber organization, cell leading edge |  | 1.24913 |
| Uchl1 | ubiquitin carboxy-terminal hydrolase L1 [Source:MGI Symbol;Acc:MGI:103149] | axon development, axon part |  | -2.34401 |
| Ulk1 | unc-51 like kinase 1 [Source:MGI Symbol;Acc:MGI:1270126] | axon development |  | -1.84794 |
| Unc13a | unc-13 homolog A [Source:MGI Symbol;Acc:MGI:3051532] | axon part |  | -1.49314 |
| Unc5a | unc-5 netrin receptor A [Source:MGI Symbol;Acc:MGI:894682] | axon development, cell leading edge |  | -1.59302 |
| Unc5d | unc-5 netrin receptor D [Source:MGI Symbol;Acc:MGI:2389364] | axon development | -0.654463 | -2.15262 |
| Ust | uronyl-2-sulfotransferase [Source:MGI Symbol;Acc:MGI:2442406] | axon development |  | -1.63322 |
| Vim | vimentin [Source:MGI Symbol;Acc:MGI:98932] | axon development, cell leading edge |  | 3.49899 |
| Vit | vitrin [Source:MGI Symbol;Acc:MGI:1921449] | cell-substrate adhesion |  | 3.071 |
| Vtn | vitronectin [Source:MGI Symbol;Acc:MGI:98940] | cell-substrate adhesion, extracellular matrix binding | 5.45535 |  |
| Vwc2 | von Willebrand factor C domain containing 2 [Source:MGI Symbol;Acc:MGI:2442987] | cell-substrate adhesion |  | -2.90498 |
| Was | Wiskott-Aldrich syndrome [Source:MGI Symbol;Acc:MGI:105059] | regulation of supramolecular fiber organization |  | 4.97577 |
| Wasf1 | WAS protein family, member 1 [Source:MGI Symbol;Acc:MGI:1890563] | regulation of supramolecular fiber organization, cell leading edge |  | -1.41666 |
| Wasf2 | WAS protein family, member 2 [Source:MGI Symbol;Acc:MGI:1098641] | regulation of supramolecular fiber organization, cell leading edge |  | 2.06921 |
| Wnt5a | wingless-type MMTV integration site family, member 5A [Source:MGI Symbol;Acc:MGI:98958] | axon development, tissue migration, neuron projection fasciculation, negative chemotaxis, morphogenesis of a branching structure | -1.48537 |  |
| Xylt1 | xylosyltransferase 1 [Source:MGI Symbol;Acc:MGI:2451073] | axon development |  | 1.77683 |
| Ywhaz | tyrosine 3-monooxygenase/tryptophan 5-monooxygenase activation protein, zeta polypeptide [Source:MGI Symbol;Acc:MGI:109484] | cell leading edge | -0.6969 | -1.76361 |
| Zfyve27 | zinc finger, FYVE domain containing 27 [Source:MGI Symbol;Acc:MGI:1919602] | axon development, axon part |  | -2.34971 |
